# Docetaxel-based induction therapy prior to radiotherapy with or without docetaxel for non-small-cell lung cancer

**DOI:** 10.1038/sj.bjc.6603115

**Published:** 2006-04-25

**Authors:** G V Scagliotti, A Szczesna, R Ramlau, F Cardenal, K Mattson, N Van Zandwijk, A Price, B Lebeau, J Debus, C Manegold

**Affiliations:** 1 Department of Clinical and Biological Sciences and Department of Radiotherapy, University of Torino, S Luigi Hospital, Regione Gonzole 10, Orbassano, Turin 10043, Italy; 2 Regional Lung Disease Hospital, Otwock, Poland; 3 Regional Lung Disease Hospital, Poznan 60569, Poland; 4 Universidad de Barcelona e Instituto Catalan de Oncologia, L’Hospitalet, Barcelona 08007, Spain; 5 Helsinki University Central Hospital, Helsinki, Finland; 6 Netherlands Cancer Institute/Antoni Van Leeuwenhoek Ziekenhuis, Amsterdam 1066, The Netherlands; 7 University of Edinburgh, Edinburgh EH8 9YL, UK; 8 Pierre et Marie Curie University, Hopital Saint-Antoine, Paris 75571, France; 9 Heidelberg University Clinic, Heidelberg 69120, Germany; 10 Department of Surgery, and Heidelberg University Medical Centre, Mannheim 68167, Germany

**Keywords:** chemoradiotherapy, taxane, docetaxel, cisplatin, non-small-cell lung cancer, concurrent therapy, radiation therapy

## Abstract

This trial aimed to assess the feasibility and tumour control of concurrent chemoradiotherapy or radiotherapy alone after docetaxel-based induction chemotherapy in locally advanced non-small-cell lung cancer (NSCLC). Patients with stage IIIA/IIIB NSCLC received two 21-day cycles of induction chemotherapy with docetaxel (85 mg m^−2^, day 1) plus cisplatin (40 mg m^−2^, days 1 and 2). Patients without disease progression on day 43 were randomised to radiotherapy (2 Gy for 5 days week^−1^; total 60 Gy) alone or with docetaxel 20 mg m^−2^ once weekly every 6 weeks. Of 108 patients who received induction chemotherapy, 104 were evaluable for response. After induction chemotherapy, the overall response rate (ORR) was 44%; 91 (88%) patients had no disease progression and 89 were subsequently randomised to local treatment. After randomised therapy, the ORR was 53% (chemoradiotherapy 58%; radiotherapy 48%). Median survival and time to progression were 14.9 and 7.8 months, respectively, for chemoradiotherapy and 14.0 and 7.5 months, respectively, for radiotherapy. The most common toxicities during induction chemotherapy and randomised therapy were grades 3–4 neutropenia and grade 3 lymphocytopenia, respectively. Docetaxel–cisplatin induction therapy followed by concurrent docetaxel and thoracic radiotherapy is a feasible treatment option, showing good clinical activity and tolerability, for locally advanced NSCLC.

Despite intensive investigation, the prognosis for patients with lung cancer, up to 87% of whom have non-small-cell lung cancer (NSCLC) at diagnosis, remains poor, with an estimated 5-year survival rate of only 15.3% (Ries *et al*, 2005). The standard treatment of locally advanced unresectable NSCLC is combined chemotherapy and thoracic radiation, based on the results of several randomised phase III trials (Schaake-Koning *et al*, 1992; Dillman *et al*, 1996; Sause *et al*, 2000). Subsequent trials have demonstrated the superiority of concurrent chemotherapy and radiotherapy over a sequential approach (Furuse *et al*, 1999; Curran *et al*, 2003; Zatloukal *et al*, 2004), although at the expense of increased toxicity, in particular severe oesophageal toxicity.

Docetaxel – a new generation taxane – has shown efficacy with acceptable toxicity in patients with NSCLC, both alone and in combination with other chemotherapeutic agents (Davies *et al*, 2003). Results of a phase II study of cisplatin plus docetaxel as induction chemotherapy before local treatment strongly support the use of this approach for patients with locally advanced resectable disease, with a complete response (CR) rate of 16% and an impressive 33-month median survival (Betticher *et al*, 2003).

Docetaxel has been shown to be an effective radiosensitiser *in vitro* (Creane *et al*, 1999; Pradier *et al*, 2001) and to act synergistically in the presence of radiation in preclinical models (Scagliotti and Turrisi, 2003). The feasibility of concurrent treatment with single-agent docetaxel and radiotherapy for unresectable advanced NSCLC was established in a series of small phase I studies (Koukourakis *et al*, 1998; Mauer *et al*, 1998), and response rates of up to 80% have been reported in phase II trials in this setting (Kim and Khuri, 2002).

Based on these encouraging outcomes, this phase II study aimed to evaluate the feasibility, efficacy and toxicity of induction chemotherapy with docetaxel plus cisplatin, followed by radiotherapy with or without weekly docetaxel, in patients with locally advanced NSCLC.

## PATIENTS AND METHODS

This prospective, randomised nonblind phase II study was conducted in nine European centres. Inclusion criteria were: age 18–75 years; histologically or cytologically proven, locally advanced unresectable, NSCLC; uni- or bidimensionally measurable disease; no previous treatment for NSCLC; World Health Organization (WHO) performance status ⩽1; ⩾12 weeks life expectancy; weight loss ⩽5% within 12 weeks of study entry; adequate haematological, hepatic, renal and respiratory function. Clinical biochemistry and haematology requirements were: platelet count ⩾100 × 10^9^ l^−1^; absolute neutrophil count (ANC) ⩾2 × 10^9^ l^−1^; haemoglobin level ⩾10 g dl^−1^; serum creatinine and bilirubin levels within the institution's normal range; serum transaminase levels ⩽2.5 × the upper normal limit (UNL); forced expiratory volume in 1 s and carbon monoxide diffusing capacity ⩾45% of the reference normal values at study entry.

Exclusion criteria included: pleural or pericardial effusion or extensive vessel invasion; a diagnosis of small-cell lung cancer; prior malignancies (except cured cervical carcinoma *in situ* or nonmelanoma skin cancer or other curatively treated cancer with no evidence of disease for ⩾5 years); conditions precluding medical follow-up and protocol compliance; history of hypersensitivity reaction to polysorbate 80; peripheral neuropathy (National Cancer Institute Common Toxicity Criteria (NCI-CTC) grade ⩾2); abnormal hepatic function (aspartate aminotransferase and/or alanine aminotransferase >1.5 × UNL associated with alkaline phosphatase >2.5 × UNL); serious comorbidities.

All patients provided written, informed consent. The study was conducted in accordance with good clinical practice guidelines and the Declaration of Helsinki.

### Treatment plan

**Figure 1:**
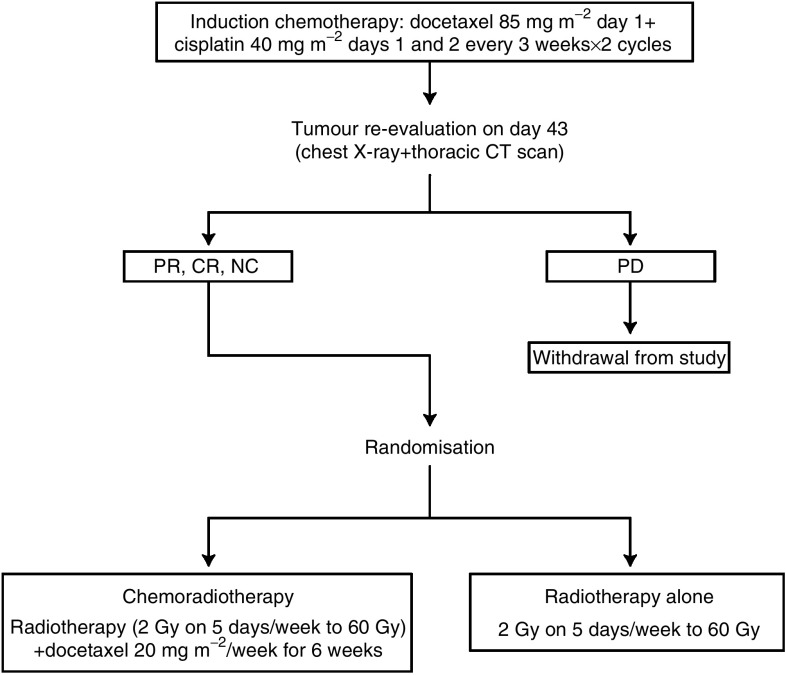
Treatment plan. CR=complete response; NC=no change; PD=progressive disease; PR=partial response.

The treatment plan is summarised in Figure 1.

#### Induction chemotherapy

Eligible patients received two 21-day cycles of induction chemotherapy comprising docetaxel 85 mg m^−2^ (1-h intravenous (i.v.) infusion) on day 1 plus cisplatin 40 mg m^−2^ (30-min i.v. infusion) on days 1 and 2. Patients received prophylactic oral dexamethasone 8 mg bid on days −1 and 1 and ondansetron 8 mg i.v. infusion on days 1 and 2. Pre- and postchemotherapy hydration was administered according to each centre's practice.

Dose modifications allowed due to toxicity were: docetaxel 75 mg m^−2^, cisplatin 40 mg m^−2^ × 2 for an ANC nadir of <0.5 × 10^9^ l^−1^ for >7 days, platelet nadir <25 × 10^9^ l^−1^, febrile neutropenia, or grades 3–4 skin toxicity or stomatitis; docetaxel 75 mg m^−2^, cisplatin 30 mg m^−2^ × 2 for grade 2 neurotoxicity, grades 3–4 nonhaematological toxicity (except anaemia), or >1 toxicity/conflicting recommendations; docetaxel 85 mg m^−2^, cisplatin 30 mg m^−2^ × 2 for nephrotoxicity grade ⩽2 during the previous cycle.

Patients were retreated on day 21 if: ANC was ⩾1.5 × 10^9^ l^−1^ and platelet count was ⩾100 × 10^9 ^l^−1^; serum creatinine was grade ⩽1 (⩽1.5 × UNL) and creatinine clearance was ⩾60 ml min^−1^; nonhaematological toxicities (except alopecia, anaemia and fluid retention) had resolved to grade ⩽1. If toxicity grade >1 persisted at day 21, treatment was delayed for up to 2 weeks. Patients with grade 3 neurotoxicity were taken off the study medication. Serum transaminase and alkaline phosphatase levels ⩽5.0–>2.5 × UNL on day 21 required a reduction in docetaxel dose to 75 mg m^−2^ for cycle 2; transaminase and alkaline phosphatase levels >5 × UNL or total bilirubin above the UNL required a treatment delay for up to 2 weeks. Patients discontinued treatment if liver toxicity persisted after dose reduction. Docetaxel was withheld in patients with moderate or severe hypersensitivity reaction until recovery from symptoms; dexamethasone 10 mg and/or diphenhydramine 50 mg infusion was recommended for moderate hypersensitivity, and epinephrine was given as needed for severe hypersensitivity. Antiemetic and antiallergic drugs were administered as needed. Prophylactic use of granulocyte- or granulocyte macrophage-colony-stimulating factor and other growth factors was not allowed during the first treatment cycle, although prophylactic dexamethasone 8 mg was given on the day before and the day of docetaxel administration.

#### Local treatment

Tumours were reevaluated on day 43 by chest X-ray and thoracic computed tomography (CT) scan, and patients with progressive disease (PD) were withdrawn from the study. The remaining patients were randomised to thoracic radiotherapy (2 Gy for 5 days each week to a total of 60 Gy using equipment that delivered megavoltage photons ⩾6 MeV) either alone or with docetaxel 20 mg m^−2^ (30-min infusion) once-weekly for 6 weeks. Radiation was administered 2–4 h after completing the docetaxel infusion (based on the European Organisation for the Research and Treatment of Cancer (EORTC) Radiotherapy Group and International Commission on Radiation Units and Measurements (ICRU) 50 recommendations (International Commission on Radiation Units and Measurements, 1993), computed tomography planning was mandatory. The radiation dose was administered to a planning target volume that included the radiologically visible primary tumour plus 1.5–2 cm margins and involved lymph nodes (including mediastinal lymph nodes >1.5 cm in their smallest diameter) plus 1–1.5 cm margins plus elective irradiation of the mediastinal lymph node regions 2–8 and the ipsilateral hilar lymph nodes. The clinical tumour volume for the primary tumour was either the pre- or postchemotherapy tumour volume, according to the single investigator's opinion. It was recommended that <30% of the total lung volume should receive >25 Gy and <50% of the total lung volume should receive >20 Gy (from November 2000, all radiotherapist investigators recommended a V20 ⩽40%, though consensus was reached that the protocol would not be amended in this respect). The heart could tolerate the tumour radiation dose if applied to <30% of its volume but could tolerate <50% of the tumour dose if applied to >50% of its volume. The spinal cord received ⩽75% of the tumour dose and ⩽15 cm of the oesophagus was included in the high-dose volume. The protocol for the radiotherapy procedure was amended after the start of enrolment (7 April 2000) in order to reduce the magnitude of the irradiated field and the potential toxicity of the treatment, following four suspected cases of treatment-related pneumonitis in the first 36 patients enrolled in the study (although in three cases the serious adverse event was later concluded to be pneumonia rather than pneumonitis). The clinical tumour volume for the primary tumour was defined in the amendment as either the prechemotherapy or the postchemotherapy tumour volume according to the single investigator's opinion; the defined inclusion of the mediastinal lymph nodes, regions 2–8 and the hilar lymph nodes coded as 10 in the clinical tumour volume for the adjacent nodal draining area; and elective irradiation of the mediastinal lymph node regions 2–8 and the ipsilateral hilar lymph nodes was removed. To assure quality control of radiotherapy, planning information, simulator films and dose distributions, including dose volume histograms, and a copy of the treatment prescription were made available for central review.

No dose modifications were planned for local treatment. In the case of toxicity, docetaxel infusion was interrupted until resolution to grade ⩽1, up to a maximum cumulative delay of 10 days. Patients were withdrawn if the schedule was interrupted for >7 consecutive days due to intercurrent illness.

### Patient evaluation

All patients underwent a full physical examination (including determination of WHO performance status, weight loss, vital signs and lung function) and clinical biochemistry tests at baseline, 3-weekly during induction chemotherapy, and again before and after local treatment. Assessments for haematology, hepatic function and toxicity were conducted at baseline, 3-weekly during induction chemotherapy (weekly for complete blood count haemoglobin, lymphocytes (with white blood cell differentials) and platelets), and weekly throughout local treatment (every 2 days in case of grade 4 or febrile neutropenia). Anteroposterior/lateral chest X-ray and thoracic CT scan (including upper abdomen scan to assess liver and adrenal gland status) were performed ⩽2 weeks and ⩽4 weeks, respectively, before starting induction chemotherapy; thoracic CT scan was repeated ⩽1 week after finishing induction chemotherapy and ⩽12 weeks after starting local treatment. Brain CT and bone scans were performed if clinically indicated. Nonmeasurable lesions were evaluated by appropriate clinical and/or radiological examination. Tissue or cytologic diagnosis was made using biopsy/brushing or bronchial aspirate obtained during fibreoptic bronchoscopy or, alternatively, transthoracic aspiration biopsy of the primary tumour. After withdrawal or completion of study treatment, patients were followed up every 3 months until PD, for a maximum of 1 year from the date of last local treatment of the last patient enrolled in the study. Any treatment-related side effects were followed until resolution.

Tumour response was assessed according to WHO criteria (WHO, 1979) (except that response to induction therapy did not require confirmation 4 weeks later). A CR was defined as the disappearance of all measurable lesions for ⩾4 weeks, a partial response (PR) as a decrease of ⩾50% of the sum of the products of the greatest perpendicular lesion diameters for ⩾4 weeks with no evidence of new lesions, and no change (NC) as a <50% decrease or <25% increase in the products of the greatest perpendicular lesion diameters with no evidence of new lesions for ⩾4 weeks. Progressive disease was defined as an increase in lesion diameter products of ⩾25% or the detection of new lesions. Time to progression (TTP) was defined as the time from the start of induction therapy until first progression or death due to PD. Survival was determined from the start of induction therapy to death from any cause.

Toxicity was assessed using NCI-CTC. Toxicities not reported in the NCI-CTC scale were graded as mild, moderate, severe or life threatening, according to MedDRA (Medical Dictionary for Regulatory Activities) 6.1. An adverse event was reported as serious if it: was fatal or life threatening; required or prolonged hospitalisation; resulted in persistent or significant disability or incapacity; was a congenital anomaly or birth defect; was an important medical event. All patients were evaluable for toxicity from the time of their first dose of study drug.

Efficacy was evaluated in all patients who were allocated to randomised therapy after induction treatment, with patients stratified according to centre and disease stage. The primary objective was overall response rate (ORR) at study end (i.e. 12 weeks from randomisation) for the intent-to-treat (ITT) population. The main secondary efficacy analyses were ORR at week 12 for the population evaluable for response to local treatment (defined as all responders to induction therapy who had received ⩾3 weekly administrations of docetaxel plus 6 weeks of radiation (chemoradiotherapy arm) or ⩾6 weeks of radiation (radiotherapy only arm), unless progression occurred in which case the outcome was described as early PD), and TTP and survival in the ITT population were determined by Kaplan–Meier analysis.

The minimum sample size was 37 evaluable patients based on a single-stage Fleming design. Combined treatment would be considered insufficiently or sufficiently promising for further study if the ORRs were ⩽30 and ⩾53%, respectively (type I error of 5% and type II error of 10%). Assuming that 10% of patients would be nonevaluable and approximately 20% would not be randomised, it was calculated that 105 patients should be enrolled.

## RESULTS

### Patients and treatment administration

Overall, 108 patients were enrolled between December 1999 and October 2001. Table 1 shows the baseline patient and tumour characteristics. Most participants had stage IIIB disease. All enrolled patients started at least one cycle of induction chemotherapy and were included in the safety population. Of these, 104 patients were evaluable for response to induction chemotherapy: the ORR was 46/104 (44%, all PRs), with NC in 45 patients (43%). Thus, 91 patients (88%) did not have PD (Table 2). Eighty-nine patients were subsequently randomised to local treatment (ITT population). Reasons for treatment discontinuation among the remaining 19 patients were: PD (*n*=10), protocol deviation (*n*=1), adverse event (*n*=2), death (*n*=3) and other (*n*=3: investigator decision, metastasis, massive decay of lesions (a contraindication for radiotherapy owing to the high risk of developing life-threatening pulmonary haemorrhage) (one patient each)).

The 89 patients comprising the ITT population were randomised to receive chemoradiotherapy (*n*=43) or radiotherapy alone (*n*=46). Of these, 22 had stage IIIA and 67 had stage IIIB disease. Patient characteristics were well balanced between the chemoradiotherapy and radiotherapy-only groups: stage IIIB disease in 74 and 76% of patients, respectively; WHO performance status of one in 79 and 78%, respectively; and squamous cell histology in 42 and 46%, respectively. Full response data after randomisation were available for all 89 patients; however, 15 patients were considered nonevaluable (nine in the chemoradiotherapy arm and six in the radiotherapy alone arm). Thus, 74 patients were considered evaluable for response to local treatment (per protocol (PP) population); the main reasons for nonevaluability were receiving <6 weeks of local treatment and lesion measured using a different method than at baseline.

The median cumulative doses of docetaxel and cisplatin administered during induction therapy were 170 mg m^−2^ (range: 84–180 mg m^−2^) and 160 mg m^−2^ (53–172 mg m^−2^), respectively (*n*=108). The median relative dose intensity was 98% for both agents (range: 53–106% for docetaxel and 53–108% for cisplatin). No chemotherapy administrations were delayed during the induction period. Five patients required docetaxel dose reduction during induction chemotherapy because of serious adverse events (SAEs): fever without infection (*n*=1), grades 3–4 infection with neutropenia (*n*=3), and grade 3 dyspnoea and vomiting (*n*=1).

During randomised therapy, the median cumulative docetaxel dose administered in the chemoradiotherapy arm was 120 mg m^−2^ (range: 20–132 mg m^−2^; *n*=41) and the median relative dose intensity was 95% (44–117%). Only one patient required docetaxel dose reduction during local treatment (from 20 to 10 mg m^−2^ for five cycles because of grade 1 oesophagitis). Chemotherapy was delayed in five patients for up to 1 week in only 6/411 cycles administered during local treatment; reasons were: dysphagia (three cycles in two patients); stomatitis (one cycle); no reason given (two cycles). Delivery of radiotherapy closely followed the planned dosage schedule in both treatment arms.

### Response to local treatment

In the ITT population, the ORR to combined induction chemotherapy and local treatment was 53% (47/89 patients; three CR, 44 PR; PP: 44/74 (59%; three CR and 41 PR)); 58% (two CR and 23 PR) after chemoradiotherapy and 48% (one CR and 21 PR) after radiotherapy alone (Table 2). The proportion of patients with no PD was 65% (two CR, 23 PR and three NC) in the chemoradiotherapy arm and 57% (one CR, 21 PR, four NC) in the radiotherapy arm. In the PP population, the ORR was 65% (two CR and 20 PR) after chemoradiotherapy and 55% (one CR and 21 PR) after radiotherapy; the proportion of patients with no PD was 71% (two CR, 20 PR and two NC) and 65% (one CR, 21 PR and four NC), respectively.

In stage IIIA patients, the ORR for chemoradiotherapy and radiotherapy was 64 and 45%, respectively, in the ITT analysis (Table 2) and 86 and 50%, respectively, in the PP analysis. In stage IIIB patients, the ORR to chemoradiotherapy and radiotherapy was 56 and 49%, respectively, in the ITT analysis and 59 and 57%, respectively, in the PP analysis. Two CR to chemoradiotherapy were achieved, irrespective of staging. One CR was seen with radiotherapy alone in a stage IIIB patient.

Overall, 23 and 31 patients in the chemoradiotherapy and radiotherapy alone arms, respectively, had a tumour relapse during the study (excluding the follow-up period). In the chemoradiotherapy arm, 13 patients had a relapse into the lung or mediastinum (three outside the field of irradiation) and nine relapsed outside this area; information on relapse location was missing for one patient. In the radiotherapy alone arm, 18 patients had a relapse into the lung or mediastinum (four outside the field of irradiation) and 10 relapsed outside this area; information on relapse location was missing for three patients.

#### Survival

**Figure 2:**
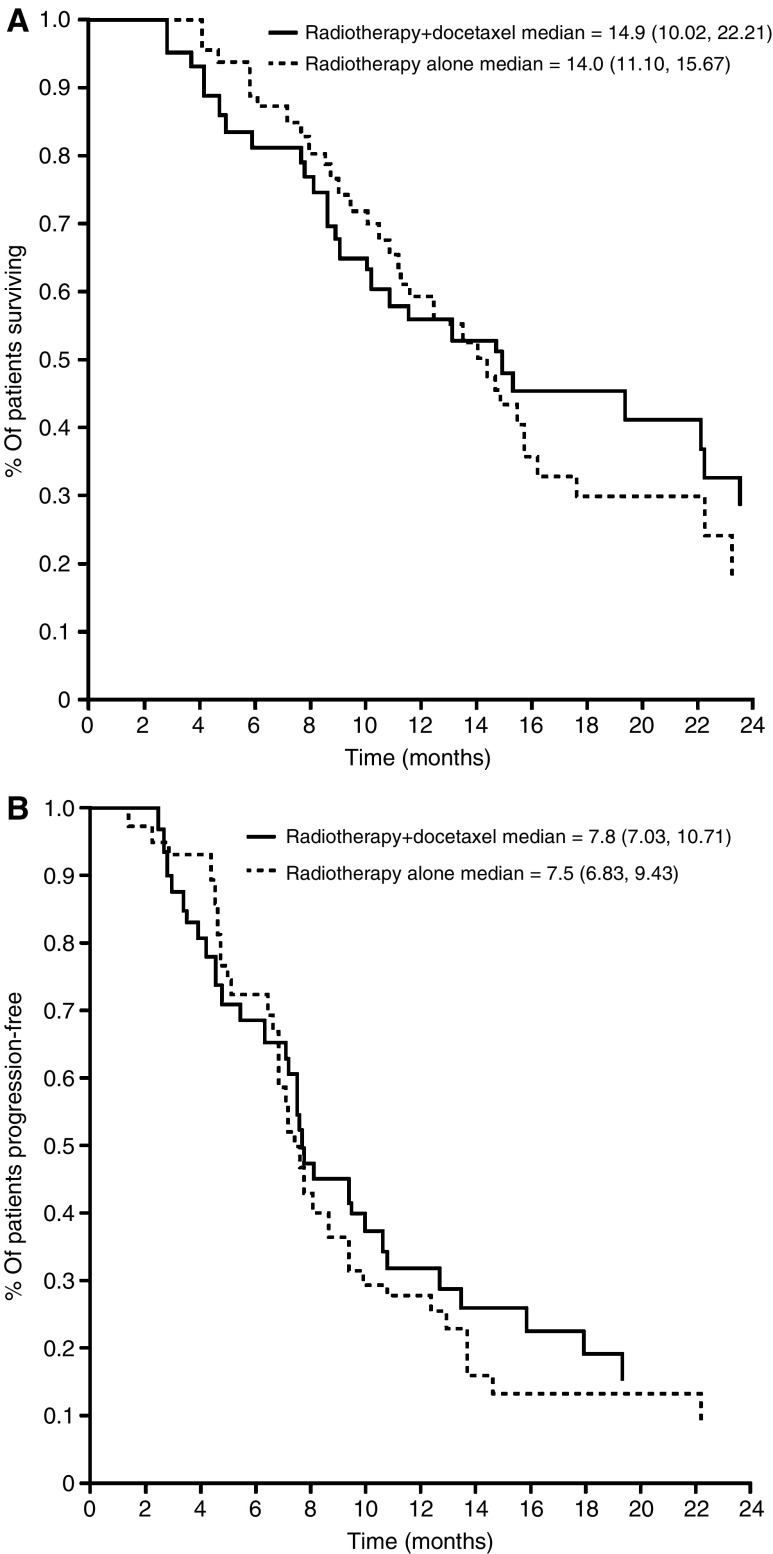
Kaplan–Meier curve of estimated (**A**) survival and (**B**) time to disease progression in patients receiving radiotherapy plus docetaxel (*n*=43) or radiotherapy alone (*n*=46) following induction chemotherapy.

The overall median survival across the ITT population was 14.6 months (95% confidence interval (CI): 11.20–16.20 months). The median survival was similar in the chemoradiotherapy and radiotherapy alone arms: 14.9 months (95% CI: 10.02–22.21 months) and 14.0 months (95% CI: 11.10–15.67 months), respectively (Figure 2A). The 1-year survival rates were also similar: 55.8% (95% CI: 39.88–70.92%) and 58.7% (95% CI: 43.23–73.00%) in the chemoradiotherapy and radiotherapy arms, respectively.

In stage IIIA patients, the 1-year survival rate was 63.6% with chemoradiotherapy and 72.7% with radiotherapy.

The median TTP was 7.6 months (95% CI: 7.03–9.43 months): 7.8 months (95% CI: 7.03–10.71 months) with chemoradiotherapy and 7.5 months (95% CI: 6.83–9.43 months) with radiotherapy alone (Figure 2B).

### Safety and toxicity

Overall, neutropenia and leucopenia were the most common NCI–CTC grades 3–4 haematological adverse events during induction chemotherapy, occurring in >20% of patients (Table 3). Lymphocytopenia and febrile neutropenia were noted much less frequently (febrile neutropenia of any grade affected only 6% of patients). Alopecia was the most frequent treatment-related nonhaematological toxicity (60 patients (56%), all grades).

Grade 3 lymphocytopenia was the most commonly reported toxicity during local treatment: 80% for chemoradiotherapy and 20% for radiotherapy alone. However, grades 3–4 infection and related adverse events occurred infrequently (e.g. infection without neutropenia: 7 and 0%, cough: 2 and 0%, dyspnoea: 5 and 7% for chemoradiotherapy and radiotherapy, respectively), showing no evidence that lymphocytopenia was associated with a higher infection risk. Alopecia, oesophagitis and fatigue (mostly grades 1–2) were the most common nonhaematological toxicities during local treatment, each affecting around 50% or more of patients in both groups (all grades). Oesophagitis, the most frequently reported grade 3 nonhaematological toxicity, only affected patients receiving chemoradiotherapy (17%) (Table 3). There were only three grade 4 events during local treatment: infection without neutropenia (one chemoradiotherapy patient) and dyspnoea (one patient per arm).

The most common treatment-related SAE of any grade reported during induction therapy was febrile neutropenia (7/108; 6%). Table 4 shows the treatment-related SAEs affecting ⩾5% of patients during the local treatment period and follow-up phase. Dysphagia/oesophagitis was more common with chemoradiation than radiotherapy alone; other treatment-related SAEs were generally similar between the arms.

In total, three patients (two chemoradiotherapy patients and one radiotherapy patient) had a SAE of pneumonitis during the local treatment period or follow-up phase: two cases were fatal (one per arm) and considered related to therapy; both occurred >30 days after the last administration of study treatment.

Overall, 11/108 patients (10%) discontinued study treatment due to toxicity: four during induction chemotherapy (one patient each due to grade 4 dyspnoea, fever without infection, fatal lung haemorrhage, sudden death possibly caused by myocardial infarction or stroke); three with chemoradiotherapy (infection without neutropenia (two patients), grade 3 dysphagia/oesophagitis (one patient)); and four with radiotherapy alone (all because of infection without neutropenia).

Eleven patients died within 30 days of the last infusion: four chemoradiotherapy patients; one radiotherapy alone patient; six nonrandomised patients. Six patients (four who received induction chemotherapy only and two in the chemoradiotherapy arm) had malignant disease recorded as the cause of death. None of the five deaths following an adverse event was considered related to study treatment; causes of death were heart failure (one chemoradiotherapy patient), sudden death of unknown cause (one chemoradiotherapy patient), grade 4 pneumonia without neutropenia (one radiotherapy-only patient (this event was initially reported as radiation-related but was subsequently revised as pneumonia unrelated to study treatment)), lung haemorrhage (*n*=1, induction chemotherapy only), and sudden death caused by possible stroke or myocardial infarction (*n*=1, induction chemotherapy only).

Sixty-four patients died >30 days after the last infusion (20 chemoradiotherapy patients, 32 radiotherapy-alone patients and 12 nonrandomised patients); most were due to PD (60 patients; 94%). Of the four resulting from SAEs, one death (radiotherapy arm) resulting from radiation pneumonitis was initially considered by the investigator to be unrelated to study treatment, but this was revised subsequently to pneumonitis probably related to study radiotherapy. A second death due to pneumonitis was also considered related to study treatment and occurred in a patient who received chemoradiotherapy and developed diffuse alveolar damage. The other two adverse-event related-deaths were considered unrelated to study treatment and occurred as a result of myocardial infarction (one chemoradiotherapy patient) and respiratory insufficiency (one radiotherapy alone patient).

## DISCUSSION

In this study, docetaxel and cisplatin (a combination noted for its activity relative to other platinum-based doublets as first-line therapy in advanced NSCLC (Fossella *et al*, 2003)) resulted in no PD (i.e. achieved PR or NC) in 88% of patients. Following induction chemotherapy, outcomes with chemoradiotherapy and radiotherapy alone were similar, although there was a trend in favour of the chemoradiotherapy group. There were two (5%) CR with chemoradiotherapy and one (2%) with radiotherapy alone; the PR rate was also higher in patients treated with chemoradiotherapy *vs* radiotherapy alone (53 *vs* 46%). As expected, stage IIIA patients gained particular benefit from concomitant chemoradiotherapy, achieving an ORR almost 20% higher than those treated with radiotherapy alone. Notably, the ORR of 58% achieved with the induction chemotherapy plus chemoradiation regimen met the criterion (ORR ⩾53%) needed to reject the hypothesis that this approach is insufficiently active to justify further investigation. Median survival times in the current study were also similar between the two arms (14.0 months with induction chemotherapy before radiotherapy alone and 14.9 months with induction chemotherapy before concurrent chemoradiation; the median survival time of 14.0 months with the sequential chemotherapy then radiotherapy-alone approach was within the range of 13.3–14.6 months seen with a sequential approach in previous phase III trials (Furuse *et al*, 1999; Curran *et al*, 2003; Fournel *et al*, 2005).

Overall, the study therapy was well tolerated. Severe febrile neutropenia was noted in a small proportion of patients in the induction phase only. No febrile neutropenia was seen after randomisation in either treatment group. Lymphocytopenia, a predicted adverse event with local radiotherapy and commonly observed when taxanes are administered with radiotherapy (Reckzeh *et al*, 1996; Robert *et al*, 1999), affected most patients who received chemoradiotherapy; however, no grade 4 cases were reported and no increase in vital or opportunistic infections was observed. Notably, our study demonstrated good overall tolerance of the chemoradiotherapy schedule used, with few toxicities reported with an incidence ⩾5%. The incidence of grade 3 oesophagitis was 17% (no grade 4), which was not over the expected rate (Mauer *et al*, 1998). Pneumonitis was reported as the principal toxicity in a recent study of concurrent two-dimensional radiotherapy (60–66 Gy) plus weekly docetaxel 20 mg m^−2^ and was considered by the authors to have adversely affected survival (Onishi *et al*, 2003). In the current study, treatment-related pneumonitis occurred to a similar extent with radiotherapy alone and chemoradiotherapy (one and two patients, respectively), and was fatal in one patient per arm.

The two 21-day cycles of induction chemotherapy in our study produced an ORR of 44%, which is similar to the 45% ORR seen in a phase II study of three cycles of the same induction chemotherapy in patients with stage IIIA (pN2) NSCLC (Manegold *et al*, 2004), although greater toxicity was seen in the latter study. This induction regimen produced a greater ORR (66%) in patients with resectable stage III (pN2) NSCLC (Betticher *et al*, 2003), likely due to the relatively low volume of disease in these patients.

Also similar to our findings, a preliminary analysis of a phase III randomised study, in which 219 nonprogressing patients with unresectable stage III NSCLC received two cycles of induction paclitaxel and carboplatin followed by radiotherapy with or without weekly paclitaxel, found a lower progression rate after chemoradiotherapy (48/109; 48%) than after radiotherapy alone (28/89; 32%) (Huber *et al*, 2003).

Consolidation docetaxel after concurrent chemoradiotherapy has been investigated as an alternative sequencing approach to induction chemotherapy. In a phase II study, stage IIIB patients received consolidation docetaxel after concurrent cisplatin, etoposide and radiotherapy (Gandara *et al*, 2003). Median progression-free and overall survival times were 16 and 26 months, respectively. The regimen was generally well tolerated, although grade 4 neutropenia was reported in 57% of patients receiving consolidation docetaxel, and was most common when the docetaxel dose was escalated to 100 mg m^−2^. It remains to be determined whether induction chemotherapy before concurrent chemoradiotherapy or concurrent chemoradiotherapy followed by consolidation chemotherapy is the most effective sequence, but the latter approach has thus far produced the longest survival times.

It is important to note that some patients with locally advanced NSCLC do not meet the tumour volume requirements when planning radiotherapy at baseline and thus cannot be selected in advance for chemoradiotherapy protocols. Induction chemotherapy might potentially rescue some patients presenting with bulky disease if a policy of encompassing the postchemotherapy tumour volume is adopted.

In conclusion, two cycles of docetaxel–cisplatin induction therapy followed by concurrent docetaxel and thoracic radiotherapy appears to represent a feasible treatment option for patients with locally advanced NSCLC. However, the results of the current study were not sufficiently compelling to design a phase III study of this approach. Instead, in a phase II study in patients with unresectable stage III NSCLC, we are currently evaluating the benefit of adding weekly cisplatin to weekly docetaxel plus concurrent radiotherapy after cisplatin–docetaxel induction therapy in one arm and before cisplatin–docetaxel consolidation chemotherapy in the second arm.

## Figures and Tables

**Table 1:** Patient characteristics at baseline (before induction chemotherapy)

**Parameter**	**Value (*n*=108)**
Median age, years (range)	59 (38–75)

*Gender, n* (%)	
Male	84 (78)
Female	24 (22)

*WHO performance status, n* (%)	
0	21 (19)
1	87 (81)

*Histology, n* (%)	
Adenocarcinoma	36 (33)
Large cell carcinoma	7 (6)
Squamous cell carcinoma	47 (44)
Other	18 (17)

*Disease stage, n* (%)	
IIIA	27 (25)
IIIB	81 (75)

*Site of disease, n* (%)	
Lung	106 (98)
Lymph nodes	96 (89)
Mediastinum	1 (1)

WHO=World Health Organization.

**Table 2:** Overall response at the end of induction chemotherapy and at study end (ITT population)

		**After local treatment**						
	**After induction chemotherapy**	**ITT population**	**Chemoradiotherapy**			**Radiotherapy alone**		
**Response, *n* (%)**	**Total (*n*=104)**	**Total (*n*=89)**	**Stage IIIA (*n*=11)**	**Stage IIIB (*n*=32)**	**Total (*n*=43)**	**Stage IIIA (*n*=11)**	**Stage IIIB (*n*=35)**	**Total (*n*=46)**
Overall response (%)	46 (44)	47 (53)	7 (64)	18 (56)	25 (58)	5 (45)	17 (49)	22 (48)
95% Confidence interval	—	—	31–89	38–74	42–73	17–77	31–66	33–63
Complete response	0	3 (3)	1 (9)	1 (3)	2 (5)	0	1 (3)	1 (2)
Partial response	46 (44)	44 (49)	6 (55)	17 (53)	23 (53)	5 (45)	16 (46)	21 (46)
No change	45 (43)	7 (8)	1 (9)	2 (6)	3 (7)	0	4 (11)	4 (9)
Progressive disease	9 (9)	19 (21)	2 (18)	5 (16)	7 (16)	6 (55)	6 (17)	12 (26)
Early progression^a^	4 (4)	8 (9)	0	4 (13)	4 (9)	0	4 (11)	4 (9)
Nonevaluable	—	8 (9)	1 (9)	3 (9)	4 (9)	0	4 (11)	4 (9)

a Progression before the first assessment of the response, that is before completion of two cycles of induction chemotherapy (6 weeks) or before completion of local treatment (3 weekly administrations of docetaxel plus 6 weeks of radiation (chemoradiotherapy arm) or at least 6 weeks of radiation (radiotherapy only arm)).

**Table 3:** Haematological and nonhaematological toxicity: NCI-CTC grades 3–4 adverse events^a^ noted in ⩾5% of patients in any treatment group

		**Randomised therapy**	
**Toxicity NCI-CTC grade**	**Induction chemotherapy (*n*=108)**	**Chemoradiotherapy^b^ (*n*=41)^c^**	**Radiotherapy alone (*n*=46)**
*Haematological, n* (%)			
Leucopenia	28 (26)	—	—
Lymphocytopenia	5 (5)^d^	33 (80)^d^	9 (20)^d^
Neutropenia	50 (46)	—	1 (2)^d^
Febrile neutropenia	7 (6)^e^	—	—

*Nonhaematological*^f^*, n* (%)			
Infection (without neutropenia)	—	3 (7)	—
Vomiting	5 (5)^d^	—	—
Oesophagitis/dysphagia	—	7 (17)^d^	—
Fatigue	3 (3)^d^	5 (12)^d^	2 (4)
Dyspnoea	1 (1)	2 (5)	3 (7)

NCI-CTC=National Cancer Institute Common Toxicity Criteria (version 2.0).

a Serious adverse events are reported separately.

b Local treatment period includes the 30 days after the last infusion for nonhaematological toxicities and the 7 days after the last infusion for haematological toxicities.

c Two patients were excluded owing to being randomised to local treatment but not receiving their randomised therapy.

d All grade 3.

e Includes all grades of febrile neutropenia.

f Alopecia graded in error as grade 3 was reported in 11 (10%) patients during induction chemotherapy, and in five (12%) and two (4%) patients in the chemoradiotherapy and radiotherapy groups, respectively, during randomised therapy. According to NCI-CTC classification, grades 3 and 4 cannot be applied to alopecia.

**Table 4:** Treatment-related serious adverse events affecting ⩾5% of patients during the local treatment period^a^ and follow-up phase

	**Number of patients (%)**		
**Toxicity NCI-CTC grade**	**Induction chemotherapy only (*n*=21)**	**Chemoradiotherapy (*n*=41)^b^**	**Radiotherapy alone (*n*=46)**
Oesophagitis/dysphagia	—	7 (17)	—
Infection without neutropenia grade 4	—	4 (10)	2 (4)
Fever without infection or neutropenia grade 4	1 (5)	3 (7)	4 (9)
Pneumonitis	—	2 (5)	—
Dyspnoea	—	2 (5)	2 (4)
Infection with neutropenia grade 4	1 (5)	2 (5)	1 (2)
Anaemia	1 (5)	—	—
Cardiac dysrhythmia	1 (5)	—	—
Reduced performance status	1 (5)	—	—
Vomiting	1 (5)	—	—

a Local treatment period includes the 30 days after the last infusion for nonhaematological toxicities and the 7 days after the last infusion for haematological toxicities.

b Two patients were excluded owing to being randomised to local treatment but not receiving their randomised therapy.
